# British Medical Students and Military Service

**Published:** 1918-07-20

**Authors:** 


### BRITISH MEDICAL STUDENTS AND MILITARY SERVICE.

We are indebted to the *British Medical Journal* for the following particulars relating to the regulations in force in the United Kingdom under Army Council Instruction No. 153 of 1918 and the National Service Instruction No. 35 of 1918, issued by the Minister of National Service.

### Army Council Instruction.

A new Army Council Instruction, No. 153 of 1918, has been issued with regard to the release of medical students serving with the Colours for the purpose of resuming their professional studies. A medical student now serving with the Colours who at the time of his enlistment was engaged in medical studies, and who (*a*) is in his third year, or (*b*) at the time of enlistment had passed the whole of the professional examination in chemistry, physics, and biology (or botany and zoology), for a medical degree or licence, is, if eligible and he so desires, to be transferred (regardless of his medical category) to Class W or W(T) of the Reserve, or discharged if ineligible for transfer to the Reserve, for the purpose of resuming his studies with a view to obtaining a medical qualification. A third-year student is defined as one who had on or before enlistment completed two years of medical study, and who can within thirty-six months complete his professional curriculum and obtain his medical degree or licence. A medical student desiring to be released from the Colours under this Instruction must apply through the usual channels, stating the date on which he wishes to be released, and undertaking to resume his studies with a view to qualifying. If a third-year medical student, he will be required to produce a certificate from the dean of his medical school (or the official corresponding to the dean), showing (1) whether he has or has not passed his professional examination in anatomy and physiology; (2) that prior to enlistment he had satisfactorily pursued his professional studies during two years, and was then actively studying with a view to qualification; and (3) that he can qualify within thirty-six months. If not a third-year student, he must produce a certificate from his dean showing that prior to enlistment he had passed the whole of the first professional examination, and that at or immediately before enlistment he was actively pursuing medical studies and had been studying as a whole-time medical student for at least six months at a recognised medical school or college. In each case the student will add to the certificate the address of the medical school at which, and the date on which, he is going to resume study. General Officers Commanding at home and abroad are directed to issue the necessary orders for bringing the provisions of this instruction before the notice of the medical students concerned, and to obtain from officers commanding units the names of men who have produced the certificate mentioned above, and who desire to be released.

### The National Service Regulations.

The Minister of National Service has issued detailed directions with regard to the "protection from military service of medical students" now in civil life (National Service Instruction No. 35 of 1918). These may be looked upon as the obverse of Army Council Instruction No. 153 of 1918, which governs the release of medical students from the ranks, just summarised. (1) A medical student who on March 5, 1918, was a full-time student at a recognised medical school, and had at that date passed his professional examination in chemistry, physics, and biology (or botany and zoology) for a medical degree or licence is not (subject to paragraphs 5, 6, 7, and 8, below) to be called up, whatever his medical category or grade, so long as he remains a full-time medical student. (2) A medical student who on March 5, 1918, was a full-time student at a recognised medical school, and furnishes to the A.D.R. of his area a certificate from the dean, or corresponding official, of his medical school that he should be able to pass his first professional examination as above on or before July 31 next, is not to be called up before July 31 next, whatever his medical category or grade. If he passes that examination by July 31 next his case will thenceforward be treated as if covered by paragraph 1. If he does not pass by that date he will forthwith be called to the Colours if otherwise available and required for service, unless he comes within the terms of paragraph 3. (3) A medical student (other than one whose case is covered, or is to be treated as if covered, by paragraph 1) who is or becomes a full-time student at a recognised medical school, and who is in Category B 2, B 3, C 2, or C 3, or is placed in Grade 3, is not (subject to paragraphs 4, 5, 6, 7, and 8) to be called up, so long as he remains a full-time student, without reference to the Director of National Service for the region. (4) A student protected under paragraph 3 who does not within twelve months of commencing his professional studies at a recognised medical school pass his first professional

(*Continued on p.* 344.)

### British Medical Students and Military Service.

(*Concluded from p.* 342.)

examination as above, will forthwith be called up if otherwise available and required for service. (5) A student protected under this Instruction who fails to pass his professional examination in anatomy and physiology within thirty-six months of commencing his professional studies at a recognised medical school will similarly be called to the Colours. (6) For protection under this Instruction a student must be enrolled in an O.T.C. and fulfil after enrolment the conditions of efficiency laid down for medical cadets. (7) Protected students delaying qualification unnecessarily, or otherwise not satisfactorily pursuing their studies, are to be referred to the Director of National Service. (8) Protection will be withdrawn from a student who has been requested in writing by the Ministry of National Service to offer himself as a surgeon probationer, R.N., and has not within twenty-one days applied for enrolment as such.

Finally the Instruction deals with formalities to be observed in the matter of certificates, and of applications to tribunals in respect of medical students not hitherto called up but now no longer protected.

